# Contextual determinants of induced abortion: a panel analysis

**DOI:** 10.1590/S1518-8787.2016050005917

**Published:** 2016-03-10

**Authors:** Mar Llorente-Marrón, Montserrat Díaz-Fernández, Paz Méndez-Rodríguez

**Affiliations:** Departamento de Economía Cuantitativa. Facultad de Economía y Empresa. Universidad de Oviedo. Oviedo, España

**Keywords:** Induced Abortion, Socioeconomic Factors, Social Vulnerability, Health Inequalities

## Abstract

**OBJECTIVE:**

Analyze the contextual and individual characteristics that explain the differences in the induced abortion rate, temporally and territorially.

**METHODS:**

We conducted an econometric analysis with panel data of the influence of public investment in health and per capita income on induced abortion as well as a measurement of the effect of social and economic factors related to the labor market and reproduction: female employment, immigration, adolescent fertility and marriage rate. The empirical exercise was conducted with a sample of 22 countries in Europe for the 2001-2009 period.

**RESULTS:**

The great territorial variability of induced abortion was the result of contextual and individual socioeconomic factors. Higher levels of national income and investments in public health reduce its incidence. The following sociodemographic characteristics were also significant regressors of induced abortion: female employment, civil status, migration, and adolescent fertility.

**CONCLUSIONS:**

Induced abortion responds to sociodemographic patterns, in which the characteristics of each country are essential. The individual and contextual socioeconomic inequalities impact significantly on its incidence. Further research on the relationship between economic growth, labor market, institutions and social norms is required to better understand its transnational variability and to reduce its incidence.

## INTRODUCTION

Voluntary pregnancy termination (VPT), or induced abortion, is a global phenomenon that responds to sociodemographic patterns, in which the characteristics of each country are essential. Its incidence is an important indicator of the frequency of unwanted pregnancies, and can point out gaps in contraceptive services and use of effective contraception.

In Europe, it is an object of discussion. Depending on the legal system in force in each country, it may constitute a punishable act or not. In Malta and Andorra, the termination of pregnancy is forbidden and punished by law, while in the Netherlands unrestricted abortion is allowed up to 24 weeks of pregnancy; in case of fetal malformation or mother’s health risks, this time limit does not apply^a^.

Most European countries opt for a time limit law that allows unrestricted abortion during a certain number of weeks. Germany, Austria, Belgium, Bulgaria, Denmark, among others, allow it at the request of the woman during the first 12 weeks of pregnancy. Romania, Netherlands, Sweden, United Kingdom and Finland extended that time limit. In Italy, the limit is 90 days and in Portugal, 10 weeks. In countries that do not have a time limit law, it is allowed only under certain assumptions, and within certain time periods. Spain, United Kingdom and Finland are governed by circumstances such as: rape, fetal malformation, danger to the mother’s physical and mental health or socioeconomic problems. Ireland has one of the most restrictive laws, and allows terminating the pregnancy only if there is a risk to the mother’s life. Poland allows it in the first 12 weeks in case of incest, rape or fetal malformation^a^.

Regarding its incidence, the European region has experienced a significant decrease in the number of VPT, from 7.7 million in 1995 to 4.2 million in 2008, which constitutes a decrease greater than 43.0%, if we consider the rate per thousand women of childbearing age^25^. This reduction is a result of the significant decline in records in former Soviet countries, between 4.0 and 6.0% per year, in contrast to the stability of Central European countries such as Denmark, Finland, and France, which maintain their records stable^b^.

The territorial distribution of VPT differs significantly. Western Europe recorded the lowest rates in the world, with 12 VPT per thousand live births. In contrast, the Eastern countries (Bulgaria, Czech Republic, Hungary, Romania, among others) have the highest estimated rates in the world, over 500 VPT per thousand live births in 2009 (Figure), although it is acknowledged that the increased use of contraceptive methods in these countries has decreased records substantially^b^.

**Figure:**
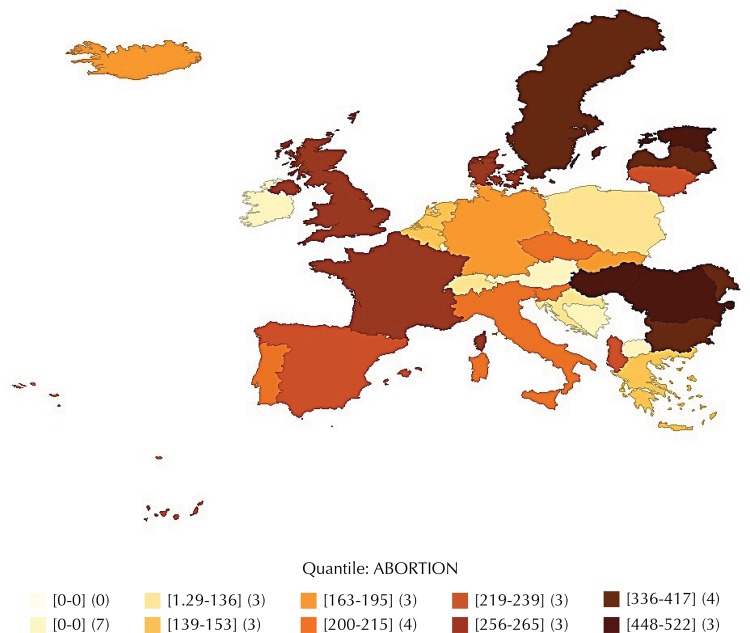
Abortions per thousand live births in Europe during 2009*.

Source: World Health Organization Regional Office for Europe. Developed from the European Health for All (HFA-DB) database. Copenhagen: WHO Regional Office for Europe; 1970 [cited 2014 Jun]. Available from: http://data.euro.who.int/hfadb/

* In brackets: intervals of abortion rates; in parentheses: number of countries by range.

As an alternative to fertility in relation to an unwanted pregnancy, VPT is associated with socioeconomic variables. Works analyzing fertility determinants are numerous, but few people have explored the unintended pregnancies, including abortion^6^ ^,^ ^10^ ^,^ ^16^. Social determinants change, and those relating to fertility and abortion are modified, with changes in the socioeconomic context of the geographical area of reference. The investigation and prevention of the causes of this situation require the knowledge of the mechanisms generating and boosting the process in each territory.

Previous studies confirm the importance of the contextual factors of a territory on VPT, such as: income level, health expenditure^4^ ^,^ ^11^, or relevance of individual determinants such as employment status, educational level, the condition of being an immigrant, age and race^4^ ^,^ ^6^ ^,^ ^9^. However, we are unaware of studies analyzing these aspects simultaneously in a number of territories, considering the characteristics and laws of each of them over a time period. In Europe, in particular, studies have been conducted in Spain^23^ ^,^ ^27^, Denmark^13^, and the United Kingdom, but a regional joint analysis has not been done. In addition, these works perform cross-sectional analyses, which disregard the temporal dimension of the phenomenon. Time and space are coordinates that jointly affect sociodemographic phenomena. Statistical modeling allowing the incorporation of both dimensions to the analysis will enable deeper analysis in studies conducted. In addition, it will allow observing factors and effects of heterogeneous behavior affecting the decision-making in the territory, and the approach to temporary effects affecting all units of study.

In this research we try to cover both aspects by analyzing, temporally and territorially, the socioeconomic determinants of VPT in 22 European countries. Our objective focuses on analyzing the contextual and individual characteristics that explain the differences in the induced abortion rate.

## METHODS

VPT econometric modelling with panel data allows seeing both the heterogeneity unobservable in the territorial sphere or temporal horizon and the analysis of its adjustment dynamics. Applying it enables analyzing the factors and effects of behavior, heterogeneous in the territory and unchangeable in time, as well as getting closer to the temporary effects common to all units of study, improving the efficiency of the econometric estimation^2^. This analysis allowed measuring the incidence of the determinants of VPT and assessing its effects, providing those politically responsible with indicators about the target population of appropriate social policy instruments to reduce its incidence. However, before proceeding to the modelling, it was necessary to check if there was spatial dependence (Moran’s index), to propose an alternative analysis method in case it were detected. Since that did not occur, a panel data econometric model was used.

The panel data econometric model is specified as:


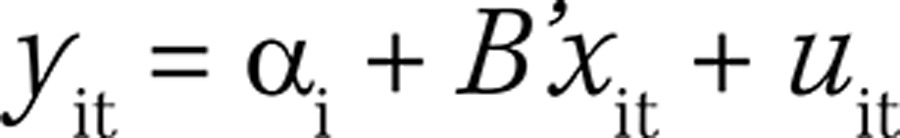


where *i* and *t* denote the cross-sectional and the temporal identifiers, respectively; *y*_it_, the dependent variable; α, vector of intercepts of n parameters; *B’*, vector of *k* explanatory variables *x*_it_ corresponding to the i-th observation at the *t* time for the *k* regressors; and u_it_, random disturbance term. The sample size with *i* = 1, …, *n* and *t* = 1, …, *T* is equal to *n* × *T*.

The interpretation of the panel data models is usually done through the analysis of its error components:


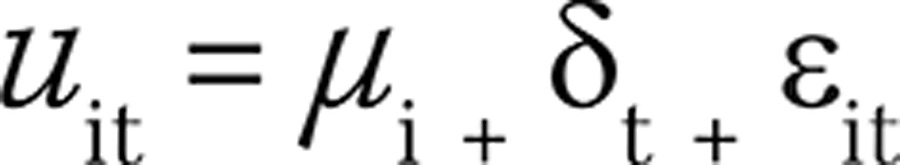


where, in addition to the random disturbance term ε_it_, *µ*_i_ collects the unobservable effects that differ only in terms of cross-sectional units, while δ_t_ collects the non-quantifiable effects exclusively linked to the time evolution. Depending on the assumptions made about *µ*_i_, the model is estimated using a fixed effects model (FEM) or random effects model (REM), and the choice between them is made based on the Hausman test^14^. In our analysis, the individual significance of variables was carried out using Student’s t-test, and the Wald test was used to assess their joint significance. The estimated model was a panel with cross-sectional fixed effects (country effects), as well as time-fixed effects; for its analysis, we used the maximum likelihood contrast for the redundancy of fixed effects. In addition to these contrasts, we assessed heteroscedasticity using the Chi-square test, autocorrelation using the D (Durbin-Watson) test, and normality of residuals using the Jarque-Bera test.

On the basis of the conceptual framework established, we analyzed: a) the influence of two essential aspects of the welfare state on VPT: public investment in health (PISALUD), measured as a percentage of gross domestic product *per capita* (GDP) invested in health by territory^6^; and income, measured by the GDP^17^; b) sociodemographic factors related to labor market and reproduction^21^: female employment rate^22^ (TEF); immigration rate^19^ ^,^ ^26^ (TMIGRACION); adolescent fertility rate^1^ ^,^ ^21^ (TFADOLESCENTE); and crude divorce rate^3^ ^,^ ^7^ (SYD).

The empirical exercise was carried out with a sample that combined information from 22 European countries for the 2001-2009 period. Germany, Belgium, Bulgaria, Croatia, Denmark, Slovakia, Slovenia, Spain, Estonia, Finland, France, Greece, Hungary, Italy, Lithuania, Norway, Netherlands, Poland, United Kingdom, Czech Republic, Romania and Sweden. The information concerning Austria, Cyprus, Ireland, Latvia, Malta and Portugal was not available in its entirety, reason why it could not be considered in the econometric analysis. The index of induced abortion was the rate of abortion per thousand live births (TIVE), which eliminates bias and allows a better approach to the subject matter, when considering only the population likely to perform this practice^9^.

National data relating to TIVE and ISALUD data were obtained from the World Health Organization Regional Office for Europe^c^. The information on the explanatory variables TEF, TFADOLESCENTE, TMIGRACION, GDP, and SYD was obtained from Eurostat^d^, and data corresponding to TFADOLESCENTE, from the Economic Commission for Europe (UNECE) Statistical Database^e^.

## RESULTS

First we conducted a spatial analysis to study whether there was a relationship of dependency between different European regions or location. Moran’s index disregarded the relationship of spatial dependence (Moran I = -0.0950867).

Then we estimated an REM (Table 1, model 1), which assumes that the correlation between unobservable effects and explanatory variables is nonexistent. The estimators were significant, and the contrast using the Wald test indicated that the model was globally significant at a level of 0.1%.

**Table 1:** Estimation of the induced abortion rate (TIVE). Europe, 2001-2009.

	Model 1	Model 2	Model 3	Model 4	Model 5
	Random effects	Fixed effects	Temporary effects	FGLS Heteroscedasticity	PCSE Heteroscedasticity
C	1070.682^a^	1445.079^a^	1.330.778	1210.787^a^	1210.787^a^
	[114.998]	[126.31]	[102.1809]	[90.8704]	[75.1461]
ISALUD	-14.2015^b^	-12.9745^b^	-14.6179^c^	-7.7007^a^	-7.7007^a^
	[5.6217]	[5.7916]	[8.4618]	[2.1917]	[2.0390]
GDP	-5.2898^a^	-7.3596^a^	-1.4085^a^	-6.1841^a^	-6.1841^a^
	[0.6890]	[0.9030]	[0.5232]	[0.5385]	[0.5684]
SYD	33.6164^c^	41.5132^b^	107.036	21.4442^a^	21.4442^a^
	[18.4693]	[19.6720]	[17.3734]	[6.4321]	[6.9256]
TEF	-24.010	-4.1021^c^	-4.2070b	-4.2911^a^	-4.2911^a^
	[1.9983]	[2.1894]	[1.7905]	[0.8220]	[0.8019]
TFADOLESCENTES	-4.8254^b^	-11.1895^a^	-8.5713^a^	-3.0200^b^	-3.0200^b^
	[2.2203]	[3.0330]	[1.2032]	[1.5305]	[1.51305]
TMIGRACION	4.3310^a^	4.3478^a^	33.661	3.6991^a^	3.6991^a^
	[1.2983]	[1.3156]	[2.4082]	[1.0368]	[0.8532]
R2	-	0.9328	0.5389	0.9638	0.9638
F	-	874.805	152.828	168.08	168.08
		[0.000]	[0.000]	[0.000]	[0.000]
WALD	19.048	23.007	334.523	56.97	-
	[0.000]	[0.000]	[0.000]	[0.000]	
WALD Heteroscedasticity	-	52357.6	-	-	-
		[0.000]			
Breusch-Pagan	409.498	-	-	-	-
	[0.000]				
Hausman test	45.009.386	-	-	-	-
	[0.000]				
Space effects	Yes	Yes	No	Yes	Yes
Time effects	No	No	Yes	No	No
Space and time effects	No	No	No	No	No
Observations	198	198	198	198	198

C: independent term; ISALUD: investment in health measured as a percentage of GDP; GDP: gross domestic product; SYD: rate of separations and divorces; TEF: female employment rate; TFADOLESCENTES: adolescent fertility rate; TMIGRACION: migration rate; FGLS: feasible generalised least squares; PCSE: panel-corrected standard error

^a^ significant at 1.0%.

^b^ significant at 5.0%.

^c^ significant at 10.0%.

Student’s t-test within brackets

There is no justification for treating the individual effects as uncorrelated with the other regressors. The application of the Hausman test allowed us to resolve this issue, since it was significant at a level of 1.0% (45.0093; p = 0.000); the REM was inconsistent.

The FEM considers that differences between territories can be captured by differences in the constant term, and is uniform over the time (model 2). The variables were significant individually, and the estimation improved substantially, R^2^ = 0.932857. The joint significance contrast of the regressors indicated that the model was significant at the level of 1.0%.

We added time dummy variables for each year, which allowed controlling by circumstances affecting territories in a given year and, therefore, reduced significant biases. We estimated the model 3, of temporary effects, and applied the maximum likelihood test for the redundancy of fixed effects, keeping the null hypothesis that the fixed effects of time are equal (1.3071; p = 0.2423), reason why we disregarded its estimation.

Therefore, the most suitable model was that of fixed effects controlled only by space (model 2). However, in the presence of heteroscedasticity (Chi-square = 52357.6), it was corrected with feasible generalized least squares (FGLS) estimators (model 4); the panel corrected standard errors (PCSE) method (model 5), which provided more precise standard errors, was used for the analysis of results. This was the model that provided the best results, with statistically significant estimators and R^2^ = 0.9638 (Table 2). Jarque-Bera’s contrast maintained the null hypothesis of normality, α = 0.9639; the maximum likelihood test for the redundancy of fixed effects indicated that these were different at a level of significance of 0.01% (83.40; p < 0.0001).

**Table 2:** Confidence intervals for regression coefficients. Model 5.

Variable	Coefficient	90%CI		95%CI		99%CI	
		
		Lower	Higher	Lower	Higher	Lower	Higher
C	1210.787	1086.505	1335.068	1062.447	1359.126	1015.026	1406.547
ISAUDE	-7.700703	-11.07337	-4.328036	-11.72624	-3.675165	-13.01310	-2.388306
GDP	-6.184140	-7.124206	-5.244075	-7.306181	-5.062100	-7.664868	-4.703413
SYD	21.44424	9.990212	32.89827	7.772976	35.11551	3.402628	39.48586
TEF	-4.291182	-5.617474	-2.964890	-5.874214	-2.708151	-6.380267	-2.202097
TFADOLESCENTES	-3.020076	-5.575805	-0.464347	-6.070535	-0.030383	-7.045687	1.005535
TMIGRACION	3.699134	5.118555	2.279712	5.393322	2.004945	5.934910	1.463357

C: independent term; ISALUD: investment in health measured as a percentage of GDP; GDP: gross domestic product; SYD: rate of separations and divorces; TEF: female employment rate; TFADOLESCENTES: adolescent fertility rate; TMIGRACION: migration rate

The results showed how higher levels of average national income and increased investments in public health reduced the incidence of induced abortion [GDP (-6.1841; p = 0.0000), ISALUD (-7.7007; p = 0.0002)]. The variability of the sociodemographic factors affecting the reproductive behavior manifested in the sign and statistical significance of sociodemographic factors peculiar to each country: civil status, female employment, migration rate and adolescent fertility rate [SYD (21.4442; p = 0.0023) TEF (-4.2911; p = 0.00001), TFADOLESCENTES (-3.0200; p = 0.0423), TMIGRACION (3.6991; p = 0.0000)].

## DISCUSSION

Although the reproductive life planning capacity is crucial for the health of women and families^24^, it remains a neglected public health issue. This study shows how the great TIVE variability observed in Europe, in the 2001-2009 period, was a consequence of socioeconomic contextual factors of each territory, and of individual socioeconomic characteristics. This work shows, for the first time, the unobservable heterogeneity of the phenomenon of induced abortion in Europe and analyzes the influence of factors that approach the level of welfare state of the territory and sociodemographic factors related to the labor market and reproduction on VPT.

The access to public health services, and its gratuitousness, is an indicator of welfare state. Previous studies showed a negative relationship between access to health care and VPT^6^ ^,^ ^7^ ^,^ ^11^. Although there is access to the public health system, the cost of contraceptives is not covered in all territories^17^, and the costs of induced abortion are borne by women. The results obtained show a negative and significant effect of the variable ISALUD (-7.7007; p = 0.0001). In *ceteris paribus c*onditions, an increase of one percentage point in health investment would generate a decrease of 3.34 of the analyzed TIVE.

The economic development impact on the fertility is ambiguous. The economic theory of human behavior^3^ interprets the reduction experienced by the fertility in developed countries as the result of a rational behavior of the family, which replaces quantity with quality of children. However, during the last decades, many countries have seen how their economic progresses have been accompanied by significant rises in fertility. This fact is mainly viewed from a certain level of economic development in which institutional changes, which improve the opportunities to reconcile paid work and life familiar, are produced^18^. The result obtained for GDP (-6.1841; p < 0.0001) reflects on the demand for children, the dominance of the income effect over the substitution effect, which means that the changes in the relationship between fertility and economic development observed in the European context^20^ are detected between VPT and economic growth.

The highest rate of induced abortion against unwanted pregnancy occurs in women who do not live as part of a couple; the greater vulnerability in this situation justifies this fact. However, decisions about family planning are modified during periods of economic turbulence^12^ ^,^ ^f^. From the theoretical consideration that children are a marital specific investment^3^, we would expect a decline in the rate of marriages to reduce fertility and increase the rate of induced abortion. The results reflect these considerations, since the variable SYD shows an effect of the regulatory role of marriage in the behavior of fertility^7^.

The difficulty of combining paid work and family responsibilities has been an important focus of investigation^5^ ^,^ ^22^. The TEF parameter estimated (-4.2911; p = 0.00001) indicates a significantly and inversely proportional effect of the variable^9^. Better female labor and socioeconomic conditions decrease the abortion rate^8^. This is associated to the fact that more educated women have higher income profiles, greater access and knowledge of contraceptive techniques, and less difficulties with unwanted pregnancies. In addition, greater stability in employment, flexible markets and laws that do not hinder part-time jobs promote the accomplishment of fertility desires, while job instability and problems associated with the labor context reduce fertility intentions^4^.

In 2010, there were 32.5 million foreigners in the European Union, a number equivalent to 6.5% of its population. The immigration of a large number of women, territorially and heterogeneously distributed, in the 2000-2010 period, substantially increased the female population of childbearing age throughout Europe.

Several studies have analyzed how the migration phenomenon is a determinant of the dependent variable^19^ ^,^ ^27^, and some suggested that the frequency of VPT in the immigrant population is higher than in the local population^26^ ^,^ ^27^. Our results show that although reproductive health programs addressing the particularities of the immigrant community are increasingly numerous, immigrants are particularly vulnerable in the European context^26^ (TMIGRACION [3.6991; p = 0.0001]). Social support, educational and economic level, knowledge and use of the health care system reduce the vulnerability of the immigrant community in the case of an unwanted pregnancy^15^.

Age is an important predictor of abortion, affecting its probability^1^ ^,^ ^13^. Induced abortion rates for those younger than 20 years reflect that, in a large number of European countries, women tend to abort when faced with unwanted pregnancies. France, Sweden, Denmark, Finland, Italy, Norway, Spain, among others, have higher incidence of induced abortions than births for those younger than 20 years^g^. In countries such as Portugal, Serbia or Romania, the incidence of abortion is lower than that of birth at all ages. The sign of the rate of adolescent fertility variable expresses this fact, and its significance shows the importance of this predictor in the incidence of abortion in Europe^21^.

The fixed effects peculiar to each country, which affect the VPT rate, approximate their facilitating or slowing effect on that rate (Table 3). The differences in the national development of the regulatory laws of induced abortion explain most of the results obtained. Some examples of the differences in such effects are: information dissemination programs within the regular processes of sexual and reproductive health care; restrictions for abortion of pregnancies over 12 weeks; sexual information programs for young women and immigrants; number and geographical distribution of family planning centers; and peculiar behaviors related to cultural and religious aspects.

**Table 3:** Estimated peculiar effects.

Country	Estimation
Poland	-596.7504
Croatia	-409.1235
Spain	-40.59503
Hungary	-3.950368
Bulgaria	-2.854302
Belgium	-25.88377
Lithuania	-248.3597
Slovenia	-201.8468
Greece	-184.5420
Czech Republic	-166.0605
Italy	-11.65880
Netherlands	121.2132
United Kingdom	131.9203
Denmark	168.3040
Romania	175.5154
Estonia	185.0873
Germany	26.69242
Sweden	373.1402
Finland	41.30129
Norway	504.1910
Slovakia	66.51944
France	97.74059

In conclusion, this study allows better understanding of the contextual determinants and individual characteristics of abortion in different European territories, of induced abortion, and of the target population for family planning activities, or sexual and reproductive education, to reduce the incidence of VPT. The study, however, has limitations due to the unavailability of micro-level data and the lack of information about VPT carried out outside the rules of each country. More research should be performed about the relationship between: economic growth, labor market, institutions, design of policies for handling working life and personal life, social norms and fertility trends, to better understand the variety of transnational patterns, and thus, reduce the incidence of induced abortion.

## Footnotes

a United Nations. Department of Economic and Social Affairs. Population Division. The Population Policy Data Bank maintained by the Population Division of the Department for Economic and Social Affairs of the United Nations Secretariat. New York; 2011 [cited 2014 Jun]. Available from: http://www.un.org/esa/population/publications/abortion/profiles.htm

b European Commission. Eurostat Statistics. Data base by themes. Luxembourg. [cited 2014 Jun]. Available from: http://ec.europa.eu/eurostat/data/database?node_code=demo_fabortord

c European Health for All database (HFA-DB). Copenhagen: WHO Regional Office for Europe; 1970 [cited 2014 Jun]. Available from: http://data.euro.who.int/hfadb/

d European Commission. Eurostat statistics. Regional Statistics. Luxembourg [cited 2014 Jun]. Available from: http://ec.europa.eu/eurostat/web/regions/data/main-tables

e United Nations. Economic Commission for Europe (UNECE). UNECE Statistical Database. Adolescent fertility. Ginebra [cited 2014 Jun]. Available from: http://w3.unece.org/PXWeb2015/pxweb/en/STAT/STAT__30-GE__02-Families_households/02_en_GEFHAdoFertility_r.px/?rxid=8ba02ce5-6180-456b-b958-1de468770e16

f Guttmacher Institute. A real-time look at the impact of the recession on women’s family planning and pregnancy decisions. New York: Institute Guttmacher; 2009 [cited 2014 Jul 14]. Available from: http://www.guttmacher.org/pubs/RecessionFP.pdf

g European Commission. Eurostat statistics. Data base by themes. Luxembourg [cited 2014 Jun]. Available from: http://ec.europa.eu/eurostat/data/database?node_code=demo_fabortord

## References

[1] Ancel PY, Lelong N, Papiernik E, Saurel-Cubizolles MJ, Kaminski M (2004). History of induced abortion as a risk factor for preterm birth in European countries: results of the EUROPOP survey. Hum Reprod.

[2] Arellano M, Bover O (1990). La econometría de datos panel. Invest Econ.

[3] Becker GS, Barro RJ (1988). A reformulation of the economic theory of fertility. Q J Econ.

[4] Begal K, Mills M (2011). The impact of subjective work control, job strain and work-family conflict on fertility intentions: a European comparison. Eur J Popul.

[5] Bloom DE, Canning D, Fink G, Finlay JE (2009). Fertility, female labor forcé participation, and the demographic dividend. J Econ Growth.

[6] Bongaarts J (2001). Fertility and reproductive preferences in post-transitional societies. Popul Dev Rev.

[7] De Irala J, Osorio A, Carlos S, Lopez-del Burgo C (2011). Choice of birth control methods among European women and the role of partners and providers. Contraception.

[8] Delgado M (1999). La evolución reciente de la fecundidad y embarazo en España: la influencia del aborto. Rev Esp Invest Sociol.

[9] Delgado M, Barrios L (2005). El aborto en España en una perspectiva internacional. Estudios Geográficos.

[10] Delgado M, Zamora López F, Barrios L (2006). Déficit de fecundidad en España: factores demográficos que operan sobre una tasa muy inferior al nivel de reemplazo. Rev Esp Invest Sociol.

[11] Finer LB, Frohwirth LF, Dauphinee LA, Singh S, Moore AM (2005). Reasons U.S. women have abortions: quantitative and qualitative perspectives. Perspect Sex Reprod Health.

[12] Finer LB, Zolna MR (2014). Shifts in intended and unintended pregnancies in the United States, 2001-2008. Am J Public Health.

[13] Hansen ML, Mølgaard-Nielsen D, Knudsen L, Keiding N (2009). Rates of induced abortion in Denmark according to age, previous births, and previous abortions. Demogr Res.

[14] Hausman JA (1978). Specification test in econometrics. Econometrica.

[15] Helstrom L, Odlind V, Zatterstrom C, Johansson M, Granath F, Correia N (2003). Abortion rate and contraceptive practices in immigrant and native women in Sweden. Scand J Public Health.

[16] Lesthaeghe R, Willems P (1999). Is low fertility a temporary phenomenon in the European Union?. Popul Dev Rev.

[17] Lete I, Dueñas JL, Martínez-Salmeán J, Parrilla JJ, Serrano I, Bermejo R (2007). Contraceptive practices and trends in Spain: 1997-2003. Eur J Obstet Gynecol Reprod Biol.

[18] Luci-Greulich A, Thévenon O (2014). Does economic advancement ‘cause’ a re-increase in fertility? An empirical analysis for OECD countries (1960-2007). Eur J Popul.

[19] Márquez-Calderón S, Rodríguez Rodríguez M (2009). Influencia de la población inmigrante en la variabilidad de la tasa de abortos entre comunidades autónomas. Gac Sanit.

[20] Myrskylä M, Kohler HP, Billari F (2009). Advances in development reverse fertility declines. Nature.

[21] Nappi RE, Lobo Abascal P, Mansour D, Rabe T, Shojai R (2014). Use of and attitudes towards emergency contraception: A survey of women in five European countries. Eur J Contracept Reprod Health Care.

[22] Orjuela-Ramírez M (2012). Aborto voluntario y actividad laboral. Reflexiones para el debate. Rev Salud Publica.

[23] Ruiz-Ramos M, Ivañez-Gimeno L, García León FJ (2012). Características sociodemográficas de la interrupción voluntaria del embarazo en Andalucía: diferencias entre población autóctona y extranjera. Gac Sanit.

[24] Ruiz Salguero MT, Cabré Plá A, Castro Martín T, Solsona Pairo M (2005). Anticoncepción y salud reproductiva en España: crónica de una revolución.

[25] Sedgh G, Singh S, Shah IH, Ahman E, Henshaw SK, Bankole A (2012). Induced abortion: incidence and trends worldwide from 1995 to 2008. Lancet.

[26] Sevoyan A, Agadjanian V (2013). Contraception and abortion in a low-fertility setting: the role of seasonal migration. Int Perspect Sex Reprod Health.

[27] Zurriaga O, Martínez-Beneito MA, Galmés Truyols A, Torne MM, Bosch S, Bosser R (2009). Recourse to induced abortion in Spain: profiling of users and the influence of migrant populations. Gac Sanit.

