# Supplementary material for: Contextual determinants of induced abortion: a panel analysis
Source: Rev Saude Publica. 2016 Mar 10;50:8. doi: 10.1590/S1518-8787.2016050005917 (PMC4794768; doi:10.1590/S1518-8787.2016050005917)
Supplement: Translation

# Translation: Determinantes contextuales del aborto inducido: un análisis panel

**Authors:** Mar Llorente-Marrón, Montserrat Díaz-Fernández, Paz Méndez-Rodríguez

**Affiliations:** Departamento de Economía Cuantitativa. Facultad de Economía y Empresa. Universidad de Oviedo. Oviedo, España

**Keywords:** Aborto Inducido, Factores Socioeconómicos, Vulnerabilidad Social, Desigualdades en la Salud

## Abstract

**OBJETIVO:**

Analizar las características contextuales e individuales que explican las diferencias en la tasa de aborto inducido, temporal y territorialmente.

**MÉTODOS:**

Se realizó un análisis econométrico, con datos panel, de la influencia de la inversión pública en salud y renta *per cápita* sobre el aborto inducido, además de una medición del efecto de factores sociales y económicos relacionados con el mercado laboral y con la reproducción: empleo femenino, inmigración, fecundidad adolescente y nupcialidad. El ejercicio empírico se realizó con una muestra de 22 países de Europa, para el periodo 2001-2009.

**RESULTADOS:**

La gran variabilidad territorial del aborto inducido fue consecuencia de factores socioeconómicos contextuales e individuales. Mayores niveles de renta nacional y de inversiones en salud pública, reducen su incidencia. Las siguientes características sociodemográficas también fueron regresores significativos del aborto inducido: empleo femenino, estado civil, migración y fecundidad adolescente.

**CONCLUSIONES:**

El aborto inducido responde a patrones sociodemográficos, en los que las peculiaridades de cada país son fundamentales. Las desigualdades socioeconómicas, a nivel individual y contextual, afectan de forma significativa su incidencia. Es necesaria más investigación acerca de relaciones entre crecimiento económico, mercado laboral, instituciones y normas sociales, para comprender mejor su variabilidad transnacional, y para poder reducir su incidencia.

## INTRODUCCIÓN

La interrupción voluntaria del embarazo (IVE), o aborto inducido, es un fenómeno global que responde a patrones sociodemográficos, en los que las peculiaridades de cada país son fundamentales. Su incidencia es un indicador importante de la frecuencia de los embarazos no deseados, y puede señalar lagunas en servicios de anticoncepción y en uso de anticonceptivos eficaces.

En Europa constituye una práctica objeto de debate. Dependiendo del ordenamiento jurídico vigente en cada país, puede constituir un acto punible o no. En Malta y Andorra, la interrupción del embarazo está prohibida y penada por ley, mientras que en Holanda se permite el aborto libre hasta las 24 semanas de gestación; en caso de malformación del feto o salud de la madre, ese plazo no aplica^a^.

La mayoría de los países europeos opta por una ley de plazos que permite el aborto libre durante un determinado número de semanas. Alemania, Austria, Bélgica, Bulgaria, Dinamarca, entre otros, lo admiten a petición de la mujer durante las 12 primeras semanas de embarazo. Rumanía, Holanda, Suecia, Reino Unido y Finlandia amplían el plazo señalado. En Italia el límite está en 90 días y en Portugal en 10 semanas. En países que no cuentan con ley de plazos, se permite solo bajo determinados supuestos, y dentro de límites temporales. España, Reino Unido y Finlandia se rigen por supuestos como: violación, malformación del feto, peligro para la salud física y psíquica de la madre o problemas socioeconómicos. Irlanda es una de legislaciones más restrictivas, y permite interrumpir el embarazo solo si hay riesgo para la vida de la madre. Polonia lo permite en las primeras 12 semanas, en caso de incesto, violación o malformación del feto^a^.

Respecto a su incidencia, la región europea ha experimentado un descenso significativo del número de IVE, pasando de 7,7 millones en 1995 a 4,2 millones en 2008, lo que configura una disminución superior al 43,0%, si consideramos la tasa por mil mujeres en edad fértil^25^. Esta reducción es consecuencia del significativo descenso de los registros en los antiguos países soviéticos, de entre 4,0 y 6,0% anual, en contraste con la estabilidad de países centroeuropeos como Dinamarca, Finlandia, o Francia, que mantienen sus registros estables^b^.

La distribución territorial del IVE difiere significativamente. El occidente europeo registra las tasas más bajas del mundo, con 12 IVE por mil nacimientos vivos. En contraste, los países del este (Bulgaria, República Checa, Hungría, Rumanía, entre otros) tienen las tasas estimadas más altas del mundo, superiores a 500 IVE por mil nacimientos vivos en 2009 (Figura), aunque debe reconocerse que la mayor utilización de métodos anticonceptivos en estos países ha disminuido sustancialmente los registros^b^.

**Figura:** Abortos por mil nacimientos vivos en Europa durante el año 2009*.

Fuente: World Health Organization Regional Office for Europe. Elaborado a partir de la base de datos European Health for All (HFA-DB). Copenhagen: WHO Regional Office for Europe; 1970 [citado 2014 jun]. Disponible en: http://data.euro.who.int/hfadb/

* Entre corchetes: intervalos de las tasas de aborto; entre paréntesis: número de países por intervalo.

Como alternativa a la fecundidad ante un embarazo no deseado, la IVE se asocia con variables socioeconómicas. Son numerosos los trabajos que estudian los determinantes de la fecundidad, pero pocos los que han profundizado en los embarazos no deseados, incluyendo el aborto^6^ ^,^ ^10^ ^,^ ^16^. Los determinantes sociales cambian, y los relativos a la fecundidad y aborto se modifican, cuando lo hace el contexto socioeconómico del ámbito territorial de referencia. La investigación y prevención de las causas de esa situación, requiere el conocimiento de los mecanismos que generan e impulsan el proceso en cada territorio.

Estudios previos constatan la importancia que tienen factores contextuales de un territorio sobre la IVE, tales como: nivel de renta, gasto sanitario^4^ ^,^ ^11^, o relevancia de determinantes individuales como status laboral, nivel educativo, condición de inmigrante, edad y raza^4^ ^,^ ^6^ ^,^ ^9^. Sin embargo, desconocemos la existencia de estudios que analicen estos aspectos simultáneamente en un conjunto de territorios, teniendo en cuenta las características y leyes propias de cada uno de ellos, a lo largo de un periodo temporal. En Europa, en concreto, se han realizado estudios en España^23^ ^,^ ^27^, Dinamarca^13^, y Reino Unido, pero no se ha hecho un análisis conjunto regional. Además, estos trabajos efectúan análisis transversales, que desconsideran la dimensión temporal del fenómeno. Tiempo y espacio son coordenadas que de forma conjunta afectan a los fenómenos de naturaleza sociodemográfica. Una modelización estadística que permita incorporar ambas dimensiones al análisis, permitirá profundizar en los estudios realizados. Adicionalmente, posibilitará la visualización de factores y efectos de comportamiento heterogéneo que condicionan la toma de decisión en el territorio, y la aproximación a los efectos temporales que afectan a todas las unidades del estudio.

Mediante esta investigación, intentamos cubrir ambos aspectos, efectuando el análisis, temporal y territorialmente, de los determinantes socioeconómicos de la IVE en 22 países de Europa. Nuestro objetivo se concreta en analizar las características contextuales e individuales que explican las diferencias en la tasa de aborto inducido.

## MÉTODOS

La modelización econométrica de la IVE con datos panel permite capturar, tanto la heterogeneidad no observable en el ámbito territorial u horizonte temporal, como el análisis de su dinámica de ajuste. Con su aplicación, es posible analizar los factores y efectos de comportamiento, heterogéneo en el territorio, e invariable en el tiempo, así como aproximarse a los efectos temporales comunes a todas las unidades del estudio, mejorando la eficiencia de la estimación econométrica^2^. Este análisis permitió medir la incidencia de los determinantes de la IVE, y evaluar sus efectos, dotando a los responsables políticos de indicadores acerca de la población objetivo de instrumentos de política social adecuados, para reducir su incidencia. No obstante, antes de proceder a la modelización, fue necesario comprobar si existía dependencia espacial (índice de Moran) para, en caso de que se detectase, plantear un método de análisis alternativo. Al no identificar dependencia espacial, se aplicó un modelo econométrico de datos panel.

El modelo econométrico de datos panel se especificó como:



donde, *i* y *t* denotan identificador transversal y temporal, respectivamente; *y*_it_, variable a explicar; α, vector de interceptos de *n* parámetros; *B’*, vector de *k* variables explicativas *x*_it_ correspondientes a la *i-ésima* observación de la unidad temporal *t* para los *k* regresores; y u_it_, término de perturbación aleatoria. El tamaño muestral con *i* = 1, …, *n* y *t* = 1, … *T* será igual a *n* × *T*.

La interpretación de los modelos de datos panel habitualmente se realiza mediante el análisis de sus componentes de error



donde, además de ε_it_ término de perturbación aleatoria, *µ*_i_ recoge los efectos no observables que difieren únicamente en términos de unidades transversales, mientras que δ_t_ los efectos no cuantificables ligados exclusivamente a la evolución temporal. Dependiendo de las hipótesis realizadas acerca de *µ*_i_, el modelo se estima mediante un modelo de efectos fijos (MEF) o aleatorios (MEA), y su elección se realiza con base en el test de Hausman^14^. En nuestro análisis, la significatividad individual de las variables se efectúo mediante el estadístico *t*-Student, y el test de Wald fue utilizado para valorar la significatividad conjunta de las mismas. El modelo estimado fue un panel con efectos fijos de secciones cruzadas (efectos de país), así como con efectos fijos de tiempo; para su análisis, se utilizó el contraste de máxima verosimilitud para la redundancia de efectos fijos. Además de estos contrastes, se valoró heterocedasticidad, estadístico Chi-cuadrado; autocorrelación, estadístico D (Durbin-Watson); y, normalidad de los residuos, test de Jarque-Bera.

Sobre la base del marco conceptual establecido, analizamos: a) la influencia de dos aspectos esenciales del estado de bienestar sobre la IVE: inversión pública en salud (ISALUD), medida como porcentaje del producto interior bruto *percápita* (PIB) invertido por territorio en salud^6^; y renta, medida por el PIB^17^; b) los factores sociodemográficos relacionados con el mercado laboral y la reproducción^21^: tasa de empleo femenino^22^ (TEF); tasa de inmigración^19^ ^,^ ^26^ (TMIGRACION); tasa de fecundidad adolescente^1^ ^,^ ^21^ (TFADOLESCENTE); y tasa bruta de divorcio^3^ ^,^ ^7^ (SYD).

El ejercicio empírico se realizó con una muestra que combinó la información de 22 países de Europa para el periodo 2001-2009. Se utilizó información relativa a: Alemania, Bélgica, Bulgaria, Croacia, Dinamarca, Eslovaquia, Eslovenia, España, Estonia, Finlandia, Francia, Grecia, Hungría, Italia, Lituania, Noruega, Países Bajos, Polonia, Reino Unido, República Checa, Rumanía y Suecia. La información relativa a Austria, Chipre, Irlanda, Letonia, Malta y Portugal no estaba disponible en su totalidad, razón por la que no pudo ser considerada en el análisis econométrico. El indicador del aborto inducido fue la tasa de aborto por mil nacimientos vivos (TIVE), que elimina sesgos y permite una mejor aproximación al objeto de estudio, al considerar únicamente la población susceptible de realizar esta práctica^9^.

Los datos nacionales relativos a la TIVE, y los datos de ISALUD, se obtuvieron de la *World Health Organization Regional Office for Europe* ^c^. La información de las variables explicativas TEF, TFADOLESCENTE, TMIGRACION, PIB y SYD se obtuvo de Eurostat^d^, y los datos correspondientes a TFADOLESCENTE, de la *Economic Commission for Europe* (UNECE) *Statistical Database* ^e^.

## RESULTADOS

En primer lugar, se efectúo un análisis espacial, con el objeto de estudiar si existía una relación de dependencia entre diferentes puntos o regiones europeas. El índice de Moran descartó la relación de dependencia espacial (Moran I = -0,0950867).

A continuación, se procedió a la estimación de un MEA (Tabla 1, modelo 1), que asume que la correlación entre efectos no observables y variables explicativas es inexistente. Los estimadores fueron significativos, y el contraste mediante el test de Wald indicó que el modelo era globalmente significativo a un nivel 0,1%.

**Tabla 1:** Estimación de la tasa de aborto inducido (TIVE). Europa, 2001-2009.

	Modelo 1	Modelo 2	Modelo 3	Modelo 4	Modelo 5
					
	Efectos aleatorios	Efectos fijos	Efectos temporales	FGLS Heteroscedasticidad	PCSE Heteroscedasticidad
C	1070,682^a^	1445,079^a^	133,0778	1210,787^a^	1210,787^a^
	[114,998]	[126,31]	[102,1809]	[90,8704]	[75,1461]
ISALUD	-14,2015^b^	-12,9745^b^	-14,6179^c^	-7,7007^a^	-7,7007^a^
	[5,6217]	[5,7916]	[8,4618]	[2,1917]	[2,0390]
PIB	-5,2898^a^	-7,3596^a^	-1,4085^a^	-6,1841^a^	-6,1841^a^
	[0,6890]	[0,9030]	[0,5232]	[0,5385]	[0,5684]
SYD	33,6164^c^	41,5132^b^	10,7036	21,4442^a^	21,4442^a^
	[18,4693]	[19,6720]	[17,3734]	[6,4321]	[6,9256]
TEF	-2,4010	-4,1021^c^	-4,2070^b^	-4,2911^a^	-4,2911^a^
	[1,9983]	[2,1894]	[1,7905]	[0,8220]	[0,8019]
TFADOLESCENTES	-4,8254^b^	-11,1895^a^	-8,5713^a^	-3,0200^b^	-3,0200^b^
	[2,2203]	[3,0330]	[1,2032]	[1,5305]	[1,51305]
TMIGRACION	4,3310^a^	4,3478^a^	3,3661	3,6991^a^	3,6991^a^
	[1,2983]	[1,3156]	[2,4082]	[1,0368]	[0,8532]
R^2^	-	0,9328	0,5389	0,9638	0,9638
F	-	87,4805	15,2828	168,08	168,08
		[0,000]	[0,000]	[0,000]	[0,000]
WALD	19,048	23,007	33,4523	56,97	-
	[0,000]	[0,000]	[0,000]	[0,000]	
WALD Heteroscedasticidad	-	52357,6	-	-	-
		[0,000]			
Breusch-Pagan	409,498	-	-	-	-
	[0,000]				
Prueba Hausman	45,009386	-	-	-	-
	[0,000]				
Efectos espacio	Sí	Sí	No	Sí	Sí
Efectos tiempo	No	No	Si	No	No
Efectos espacio y tiempo	No	No	No	No	No
Observaciones	198	198	198	198	198

C: término independiente; ISALUD: inversión en salud medido como porcentaje del PIB; PIB: producto interior bruto; SYD: tasa de separaciones y divorcios; TEF: tasa de empleo femenino; TFADOLESCENTES: tasa de fecundidad en adolescentes; TMIGRACION: tasa de migración; FGLS: mínimos cuadrados generalizados factibles; PCSE: errores estándar corregidos para panel

^a^ significativo al 1,0%.

^b^ significativo al 5,0%.

^c^ significativo al 10,0%.

Estadístico *t*-Student entre corchetes

No hay justificación para tratar los efectos individuales como no correlacionados con los otros regresores. La aplicación del test de Hausman nos permitió resolver esta cuestión, dado que fue significativo a un nivel del 1,0% (45,0093; p = 0,000); el MEA resultó inconsistente.

El MEF considera que las diferencias entre territorios pueden captarse por diferencias en el término constante, y es uniforme a lo largo del tiempo (modelo 2). Las variables fueron significativas individualmente, y la estimación mejoró sustancialmente, R^2^ = 0,932857. El contraste de significatividad conjunta de los represores indicó que el modelo era significativo al nivel del 1,0%.

Fueron agregadas variables *dummies* temporales para cada año, que permitieron controlar por circunstancias que afectaron a los territorios en un año dado y, por lo tanto, redujeron sesgos importantes. Se estimó el modelo 3, de efectos temporales, y se aplicó el test de máxima verosimilitud para la redundancia de los efectos fijos, manteniendo la hipótesis nula de que los efectos fijos de tiempo son iguales (1,3071; p = 0,2423), razón por la que se prescindió de su estimación.

Por consiguiente, el modelo más adecuado fue el de efectos fijos, controlado solo por el espacio (modelo 2). No obstante, ante la presencia de heteroscedasticidad (Chi-cuadrado = 52357,6), se corrigió con estimadores de mínimos cuadrados generalizados factibles (FGLS), (modelo 4); el método de errores estándar corregidos para panel (PCSE) (modelo 5) que presentó errores estándar más precisos, fue el utilizado para el análisis de resultados. Este modelo fue el que mejores resultados proporcionó, con estimadores estadísticamente significativos y R^2^ = 0,9638 (Tabla 2). El contraste de Jarque-Bera mantuvo la hipótesis nula de normalidad, α = 0,9639; el test de máxima verosimilitud para la redundancia de los efectos fijos, indicó que estos eran diferentes a un nivel de significatividad del 0,01% (83,40; p < 0,0001).

**Tabla 2:** Intervalos de confianza para los coeficientes de regresión. Modelo 5.

Variable	Coeficiente	IC90%		IC95%		IC99%	
		
		Inferior	Superior	Inferior	Superior	Inferior	Superior
C	1210,787	1086,505	1335,068	1062,447	1359,126	1015,026	1406,547
ISAUDE	-7,700703	-11,07337	-4,328036	-11,72624	-3,675165	-13,01310	-2,388306
PIB	-6,184140	-7,124206	-5,244075	-7,306181	-5,062100	-7,664868	-4,703413
SYD	21,44424	9,990212	32,89827	7,772976	35,11551	3,402628	39,48586
TEF	-4,291182	-5,617474	-2,964890	-5,874214	-2,708151	-6,380267	-2,202097
TFADOLESCENTES	-3,020076	-5,575805	-0,464347	-6,070535	-0,030383	-7,045687	1,005535
TMIGRACION	3,699134	5,118555	2,279712	5,393322	2,004945	5,934910	1,463357

C: término independiente; ISALUD: inversión en salud medido como porcentaje del PIB; PIB: producto interior bruto; SYD: tasa de separaciones y divorcios; TEF: tasa de empleo femenino; TFADOLESCENTES: tasa de fecundidad en adolescentes; TMIGRACION: tasa de migración

Los resultados mostraron cómo mayores niveles de renta media nacional, y mayores inversiones en salud pública, reducían la incidencia del aborto inducido [PIB (-6,1841; p = 0,0000), ISALUD (-7,7007; p = 0,0002)]. La variabilidad de los factores sociodemográficos que afectaron el comportamiento reproductivo, se manifestó en el signo e importancia estadística de factores sociodemográficos propios de cada país: empleo femenino, estado civil, tasa de migración y fecundidad adolescente [SYD (21,4442; p = 0,0023) TEF (-4,2911; p = 0,00001), TFADOLESCENTES (-3,0200; p = 0,0423), TMIGRACION (3,6991; p = 0,0000)].

## DISCUSIÓN

Aunque la capacidad de planificación de la vida reproductiva es fundamental para la salud de mujeres y familias^24^, sigue siendo una cuestión descuidada en salud pública. Este estudio muestra cómo la gran variabilidad de TIVE observada en Europa, en el periodo 2001-2009, fue consecuencia de factores socioeconómicos contextuales de cada territorio, y de características socioeconómicas individuales. El trabajo capta, por primera vez, la heterogeneidad no observable del fenómeno del aborto inducido en Europa, y analiza la influencia de factores que aproximan el nivel del estado de bienestar del territorio, y de factores sociodemográficos relacionados con el mercado laboral y la reproducción, sobre la IVE.

El acceso a los servicios públicos de salud, y su gratuidad, es un indicador del estado del bienestar. Estudios previos muestran una relación negativa entre acceso a la atención sanitaria e IVE^6^ ^,^ ^7^ ^,^ ^11^. Aunque hay acceso a la sanidad pública, el coste de los anticonceptivos no está cubierto en todos los territorios^17^, y los gastos de aborto inducido corren a cargo de la mujer. Los resultados obtenidos muestran un efecto negativo y significativo de la variable ISALUD (-7,7007; p = 0,0001). En condiciones *caeteris paribus*, un incremento unitario de un punto porcentual en inversión en salud, generaría un descenso de 3,34 unidades de la TIVE analizada.

El impacto del nivel de desarrollo económico sobre la fecundidad es ambiguo. La teoría económica del comportamiento humano^3^ interpreta la reducción experimentada por la fecundidad en los países desarrollados, como el resultado de un comportamiento racional de la familia que sustituye cantidad, por calidad de hijos. No obstante, durante las últimas décadas, muchos países han visto cómo sus avances económicos han estado acompañados de repuntes significativos de la fecundidad. Este hecho se visualiza fundamentalmente a partir de un determinado nivel de desarrollo económico, en el que se producen cambios institucionales que mejoran las oportunidades de conciliar trabajo remunerado y vida familiar^18^. El resultado obtenido para PIB (-6,1841; p < 0,0001), refleja en la demanda de hijos el dominio del efecto renta sobre el efecto sustitución, lo que significa que los cambios observados dentro del contexto europeo, en la relación entre fecundidad y desarrollo económico^20^, se detectan entre IVE y crecimiento económico.

La mayor tasa de aborto inducido ante embarazos no deseados se presenta en mujeres que no conviven en pareja; la mayor vulnerabilidad en esta situación justifica este hecho. No obstante, las decisiones sobre planificación familiar se modifican durante períodos de turbulencia económica^12^ ^,^ ^f^. A partir de la consideración teórica de que los niños son una inversión específica marital^3^, cabría esperar que un descenso en la tasa de matrimonios redujese la fecundidad, y aumentase la tasa de abortos inducidos. Los resultados reflejan estas consideraciones, ya que la variable SYD visualiza una acción del papel regulador de la nupcialidad en el comportamiento de la fecundidad^7^.

La dificultad de combinar trabajo remunerado y responsabilidades familiares, ha sido un foco importante de investigación^5^ ^,^ ^22^. La estimación del parámetro TEF (-4,2911; p = 0,00001) obtenida, indica un efecto significativo e inversamente proporcional de la variable^9^. Mejores condiciones socioeconómicas y laborales femeninas disminuyen la tasa de aborto.^8^ Este hecho se asocia a que las mujeres con mayor nivel de instrucción presentan perfiles de renta superior, mayor accesibilidad y conocimiento de técnicas anticonceptivas, y menores dificultades ante la materialización de embarazos no deseados. Adicionalmente, mayor estabilidad en el empleo, mercados flexibles y leyes que no penalizan el empleo a tiempo parcial, favorecen la materialización de los deseos de fecundidad, mientras que inestabilidad laboral y problemas asociados al contexto laboral, reducen las intenciones de fecundidad^4^.

En 2010, había 32,5 millones de extranjeros en la Unión Europea, equivalentes al 6,5% de su población. La inmigración de un elevado número de mujeres, distribuidas territorialmente de forma heterogénea, en el periodo 2000-2010, incrementó sustancialmente la población femenina en edad fértil en todo el contexto europeo.

Varios estudios analizaron cómo el fenómeno migratorio constituye un determinante de la variable a explicar^19^ ^,^ ^27^, y algunos sugirieron que la frecuencia de IVE en la población inmigrante es mayor que en la población nacional^26^ ^,^ ^27^. Nuestros resultados muestran que aunque son cada vez más numerosos los programas de salud reproductiva que abordan las particularidades del colectivo inmigrante, este es especialmente vulnerable en el contexto europeo^26^ (TMIGRACION [3,6991; p = 0,0001). Apoyo social, nivel educativo y económico, conocimiento y uso del sistema de salud reducen la vulnerabilidad del colectivo inmigrante ante un embarazo no deseado^15^.

La edad es un importante predictor del aborto, ya que la probabilidad de este varía de acuerdo a ella^1^ ^,^ ^13^. Las tasas de aborto inducido para las menores de 20 años reflejan que, en un gran número de países europeos, las mujeres tienden a abortar ante embarazos no deseados. Francia, Suecia, Dinamarca, Finlandia, Italia, Noruega, España, entre otros, presentan mayor incidencia de aborto inducido, que de nacimientos, para las menores de 20 años^g^. En países como Portugal, Serbia o Rumanía, la incidencia de aborto es inferior a la de nacimiento en todas las edades. El signo de la variable tasa de fecundidad en adolescentes manifiesta este hecho, y su significatividad muestra la importancia de este predictor en la incidencia del aborto en Europa^21^.

Los efectos fijos, propios de cada país, que afectan a la tasa de IVE, aproximan su efecto dinamizador o ralentizador sobre la misma (Tabla 3). Las diferencias en el desarrollo nacional de las leyes reguladoras del aborto inducido, explican la mayor parte de los resultados obtenidos. Algunos ejemplos de las disparidades en dichos efectos son: programas de difusión de información dentro de los procesos regulares de atención sobre salud sexual y reproductiva; limitaciones para practicar abortos de más de doce semanas de gestación; programas de información sexual al colectivo de mujeres jóvenes e inmigrantes; número y distribución geográfica de centros de planificación familiar; y comportamientos propios, relacionados con aspectos culturales y religiosos.

**Tabla 3:** Efectos propios estimados.

País	Estimación
Polonia	-596,7504
Croacia	-409,1235
España	-40,59503
Hungría	-3,950368
Bulgaria	-2,854302
Bélgica	-25,88377
Lituania	-248,3597
Eslovenia	-201,8468
Grecia	-184,5420
República Checa	-166,0605
Italia	-11,65880
Países Bajos	121,2132
Reino Unido	131,9203
Dinamarca	168,3040
Rumanía	175,5154
Estonia	185,0873
Alemania	26,69242
Suecia	373,1402
Finlandia	41,30129
Noruega	504,1910
Eslovaquia	66,51944
Francia	97,74059

Para concluir, este estudio permite conocer mejor los determinantes contextuales y las características individuales del aborto en los distintos territorios europeos. Proporciona resultados que permiten comprender mejor la variabilidad del aborto inducido, y proporciona un mejor conocimiento de la población objetivo de acciones de planificación familiar, o de educación sexual y reproductiva, con el objeto de reducir su incidencia. El estudio, no obstante, presenta limitaciones consecuencia de la no disponibilidad de datos a nivel micro y la no existencia de información acerca de la IVE efectuada fuera de la normativa de cada país. Es necesaria más investigación acerca de las relaciones entre: crecimiento económico, mercado laboral, instituciones, diseño de políticas de conciliación de la vida laboral, normas sociales y tendencias de fertilidad, para comprender mejor la variedad de patrones transnacionales, y poder así, reducir la incidencia del aborto inducido

## Footnotes

a United Nations. Department of Economic and Social Affairs. Population Division. The Population Policy Data Bank maintained by the Population Division of the Department for Economic and Social Affairs of the United Nations Secretariat. New York; 2011 [citado 2014 jun]. Disponible en: http://www.un.org/esa/population/publications/abortion/profiles.htm

b European Commission. Eurostat Statistics. Data base by themes. Luxembourg. [citado 2014 jun]. Disponible en: http://ec.europa.eu/eurostat/data/database?node_code=demo_fabortord

c European Health for All database (HFA-DB). Copenhagen: WHO Regional Office for Europe; 1970 [citado 2014 jun]. Disponible en: http://data.euro.who.int/hfadb/

d European Commission. Eurostat statistics. Regional Statistics. Luxembourg [citado 2014 jun]. Disponible en: http://ec.europa.eu/eurostat/web/regions/data/main-tables .

e United Nations. Economic Commission for Europe (UNECE). UNECE Statistical Database. Adolescent fertility. Ginebra [citado 2014 jun]. Disponible en: http://w3.unece.org/PXWeb2015/pxweb/en/STAT/STAT__30-GE__02-Families_households/02_en_GEFHAdoFertility_r.px/?rxid=8ba02ce5-6180-456b-b958-1de468770e16

f Guttmacher Institute. A real-time look at the impact of the recession on women’s family planning and pregnancy decisions. New York: Institute Guttmacher; 2009 [citado 2014 jul 14]. Disponible en: http://www.guttmacher.org/pubs/RecessionFP.pdf

g European Commission. Eurostat statistics. Data base by themes. Luxembourg [citado 2014 jun]. Disponible en: http://ec.europa.eu/eurostat/data/database?node_code=demo_fabortord
